# Misconceptions Regarding the Role of Introgression in the Origin of *Oryza sativa* subsp. *indica*

**DOI:** 10.3389/fpls.2018.01750

**Published:** 2018-11-29

**Authors:** Peter Civáň, Terence A. Brown

**Affiliations:** School of Earth and Environmental Sciences, Manchester Institute of Biotechnology, University of Manchester, Manchester, United Kingdom

**Keywords:** rice, domestication, gene flow, population genomics, selective sweep

In the study of crop origins, it is generally assumed that directional and purifying selection is the major force employed during domestication. This selection exerted through crop management practices creates local depressions of nucleotide diversity in the genomic regions surrounding the alleles that are advantageous for cultivation. Such local depressions of diversity often span 10^4^ − 10^6^ bp, depending on the level of linkage disequilibrium, and are called “selective sweep regions.” Due to the scale and severity of the diversity reduction, selective sweeps are relatively easy to detect and are often used as cues in the search for domestication-related genes (e.g., Tian et al., 2009; Jordan et al., 2015; Pankin et al., 2018). Large fractions of crop genomes are often so diverse and dynamic (in terms of recombination) that it is difficult to untangle their genealogical origins, so selective sweep regions are proving crucial for our understanding of the domestication process. This is particularly true for Asian cultivated rice (*Oryza sativa* L.), for which views on domestication remain controversial.

Genome-wide scans have repeatedly revealed unique diversity patterns in the three groups of *O. sativa*—*indica, japonica*, and *aus*—indicating their generally different demographic histories (Zhao et al., 2011; Huang et al., 2012; Civán et al., 2015). Each of these rice groups has a set of putative selective sweep regions presumably resulting from selection imposed during domestication. Many of these selective sweeps are group-specific, i.e., the regions are under selection only in one group, while very few of them coincide and carry identical haplotypes in two or all three groups (Civán et al., 2015). The outstanding question of Asian rice domestication concerns the genealogical history of the genes in those shared selective sweeps. These genes are uniform in all cultivated rice and include a few recessive alleles functionally related to domestication—namely *sh4* (causing non-shattering of seeds at maturity—Li et al., 2006), *prog1* (causing erect growth—Tan et al., 2008), and perhaps also *laba1* (causing short and barbless awns—Hua et al., 2015—although this trait is not fixed in all *japonica*) and *rc* (causing white pericarp and reduction of seed dormancy—Sweeney et al., 2006, 2007; Gu et al., 2011—although two different *rc* mutations underlie this phenotype in *aus*). Currently, there are two competing hypotheses regarding the genealogy of these domestication alleles: (i) the alleles existed in different wild populations prior to domestication and were selected multiple times from standing variation in independent domestication processes (Civán and Brown, 2017, 2018a); (ii) the alleles were selected and fixed in one cultivated group (*japonica* being the usual choice) and subsequently transferred to other (pre)domesticated groups by introgressive hybridization (e.g., Huang et al., 2012; Choi et al., 2017; Choi and Purugganan, 2018). Resolution of this problem currently seems to be the decisive point in the long-standing debate of single vs. multiple domestications of Asian rice. However, since both scenarios are expected to leave similar signatures in the cultivated genomes, it is inherently difficult to decipher the correct answer.

In 2015, we published an analysis of rice genomic data that indicated independent and geographically separate domestications of *japonica, indica*, and *aus* (Civán et al., 2015). Our conclusions were controversial as they contradicted a previous, high-profile analysis of the same dataset (Huang et al., 2012) and implied that gene flow played only an insignificant role in the emergence of the non-*japonica* groups. Despite the controversy and wide persistence of the introgression hypothesis in the scientific literature, only recently has an attempt been made to demonstrate that our approach and conclusions are incorrect. Choi and Purugganan (2018) once again reanalyzed the genomic dataset of Huang et al. (2012) and employed the approach for analysis of putative selective sweeps that had previously led us to conclude independent domestications. In contrast to our conclusions, Choi and Purugganan (2018) claim the results support a single *de novo* domestication of Asian rice followed by transfer of domestication alleles to other wild populations by introgression. How is it possible that two studies stemming from the same dataset and employing a similar methodological approach can reach such contrasting conclusions?

There are a few technical differences that distinguish the Civán et al. (2015) and Choi and Purugganan (2018) studies—e.g., the latter study used state-of-the art tools for genotype reconstruction from low-coverage data, which allowed narrower genomic windows to be examined. Nonetheless, following genotype reconstruction, diversity scans, and neighbor-joining tree construction, Choi and Purugganan (2018) focused their attention on three co-located low-diversity genomic regions (CLDGRs) containing the domestication-related genes *Sh4, Prog1*, and *Laba1*. These three CLDGRs were also identified and analyzed in our study (CLDGR15 for *Laba1*, CLDGR16 for *Sh4*, CLDGR21 for *Prog1*; see Supplementary Figures 3l, 3m, and 3r, respectively, in Civán et al., 2015), which shows that the contrast between the two studies does not stem from technical differences and their impact on sweep detection, but rather from distinct interpretation of very similar results (see Figures 1A–C).

**Figure 1:**
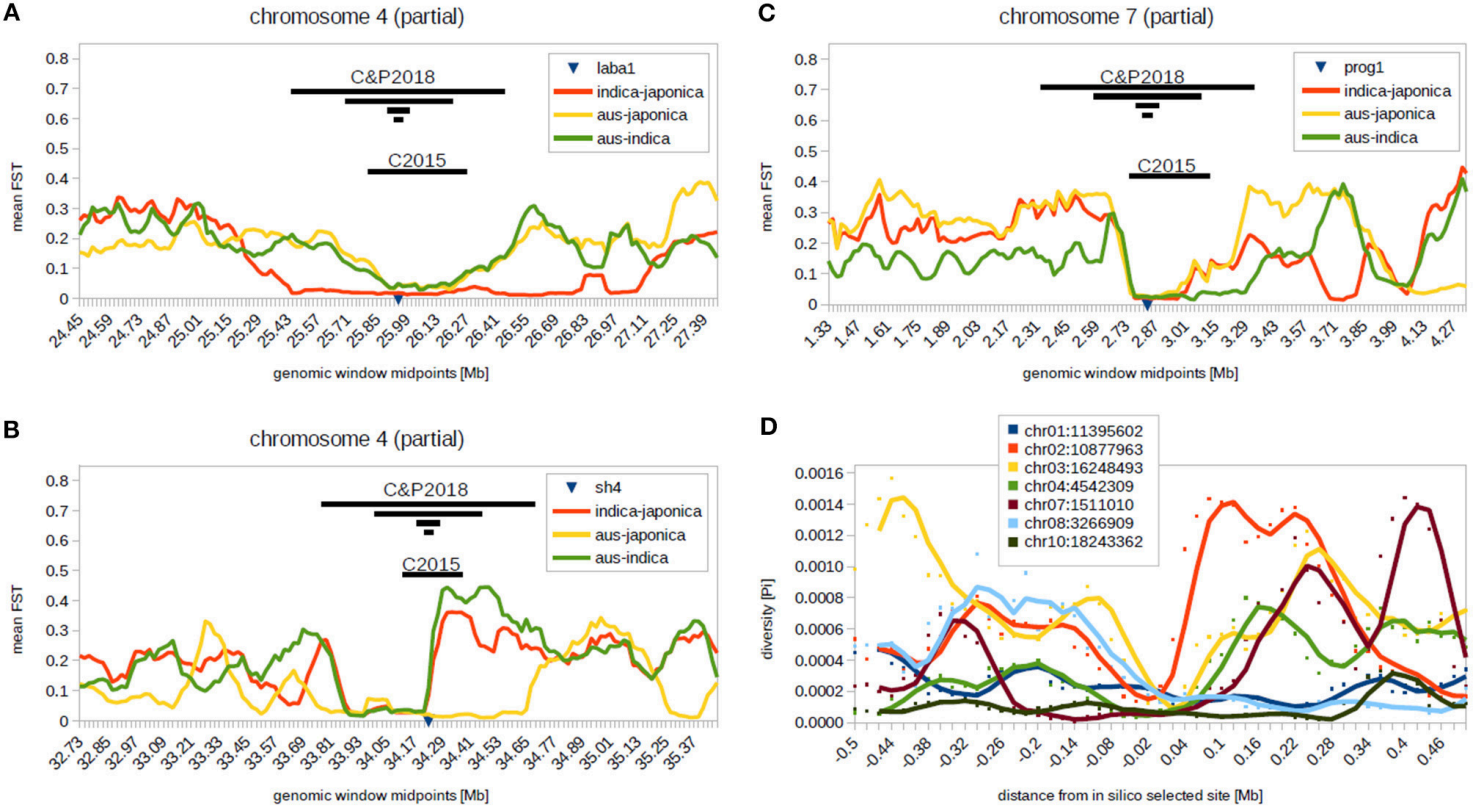
Genomic regions containing the *Laba1* **(A)**, *Sh4* **(B)**, and *Prog1* **(C)** genes. Genomic windows analyzed by Civán et al. (2015) and Choi and Purugganan (2018) are indicated with black bars. **(D)** Artificial “selective sweeps” produced by *in silico* selection of *indica* accessions carrying a minor allele in the center of the shown regions. Diversity landscapes surrounding the selected site often resemble real selective sweeps. Graphs were produced using the 29 million biallelic SNP dataset (3,000 rice genome project, 2014) downloaded from ^1^. The bed file was converted into vcf format using PLINK v1.90 (S. Purcell and C. Chang ^2^; Chang et al., 2015), selecting 283 *indica*, 154 tropical and temperate *japonica* and 124 *aus* individuals. Pairwise F_ST_ and nucleotide diversity values were calculated with VCFtools 0.1.15 (Danecek et al., 2011), using 100 kb window size and 20 kb sliding steps (physical length of the IRGSP-1.0 genome assembly).

So how did Choi and Purugganan (2018) reach the conclusion of a single *de novo* domestication of Asian rice followed by transfer of domestication alleles by introgression? The authors based their conclusion on the observation that in each of the neighbor-joining trees for the three CLDGRs carrying the *Sh4, Prog1*, and *Laba1* genes, all the domesticated rice accessions clustered together, displaying what they describe as monophyletic relationships. However, their trees are not monophyletic. For each of these genes, at each of the examined genomic window sizes, the recovered topology is paraphyletic (Figures 1B, 2A–C in Choi and Purugganan, 2018). This is a crucial distinction, because while a monophyletic *O. sativa* clade would indeed indicate a single origin for the given genomic region in *O. sativa*, a paraphyletic group has no such implication. In each of those trees, the group containing *O. sativa* also contains many *Oryza rufipogon* genotypes: e.g., with the shortest, 40 kb, windows shown in their Figure 1B there are >100 *O. rufipogon* genotypes in each of the *O. sativa* groups for the *Sh4* and *Prog1* regions, and there are ~70 such genotypes in the *Laba1* region. The paraphyly within these clusters in fact suggests that cultivated rice obtained the examined regions from multiple *O. rufipogon* individuals. This could have occurred during a single domestication, but equally could have occurred during multiple domestication episodes. The recent observations of multiple haplotypes at domestication-related loci in *O. sativa*, some of which are unshared among *indica* and *japonica* (Civán and Brown, 2017; Wang et al., 2018), further indicates that the paraphyletic trees in Choi and Purugganan (2018) should be interpreted as evidence of domestication from multiple wild lineages.

Importantly, the scarcity of the uniform sweeps (i.e., monophyletic CLDGRs) is only one part of the argument that led us to conclude that there were three independent domestications of Asian rice. Equally important is the abundance of the group-specific selective sweeps (i.e., regions under selection in only one group). Group-specific selective sweeps were detected in the original study (Huang et al., 2012) and by us (Civán et al., 2015), and although Choi and Purugganan (2018) do not mention them, it is implied that they detected them also. Since domestication can be viewed as a long-term selection experiment, signatures of group-specific selection are likely to be the signatures of separate domestication processes. Whether the group-specific selective sweeps represent initial domestications of multiple different gene pools, or appeared later as a result of post-domestication divergence of a single crop, is not explored by Choi and Purugganan (2018). Hence, their conclusion that “*de novo* domestication appears to have occurred only once” was reached without considering all the available evidence.

Although Choi and Purugganan (2018) fail to interpret their trees as paraphyletic, they are aware that domestication-related haplotypes are frequently found in wild rice. Instead of interpreting these as ancestral variants that were selected during domestication(s), they suggest a somewhat non-parsimonious explanation—that the domestication variants evolved during domestication and were subsequently transferred to wild rice by crop-to-wild gene flow. In support of this hypothesis, they mention the recent paper by Wang et al. (2017), who claim that “most modern wild rice is heavily admixed with domesticated rice” and conclude that *O. rufipogon* represents a hybrid swarm. Even though we agree that some level of gene flow is likely to have occurred in this direction, we are convinced that the scale of this problem is greatly exaggerated. Wang et al. (2017) reported a very high degree of genetic relatedness between wild rice and domesticated rice subgroups and subsequently reached the hybrid swarm conclusion by performing (i) a statistical test of correlation between genetic and geographic distances; and (ii) analysis of genetic diversity in a *Sh4*-containing sweep. However, as we show below, the results of both tests were misinterpreted.

Wang et al. (2017) correctly point out that the genetic similarity of wild and domesticated rice can be due to ancestor–descendant relationship or to gene flow. They propose that a strong correlation between genetic and geographic distances in crop-wild pairs would support the second alternative. Subsequently, they report “a highly significant correlation (ρ = 0.15, *P* < 2.2 × 10^−16^)” and conclude that this signals gene flow. This is, however, a misleading interpretation of the statistical test. In fact, although the *P*-value indicates that the result is extremely unlikely to be due to chance (thanks to a very large number of comparisons), the detected correlation is still very weak (*r* = 0.15). Moreover, the correlation plots (Supplementary Figures S9, S10 in Wang et al., 2017) clearly show that this weak correlation is due to geographically distant pairs that are genetically dissimilar (the top right quarter of the plots), and not due to geographically close pairs that are genetically similar. This implies that geographically close pairs do not display correlation between genetic and geographic distances, which means that gene flow between wild and domesticated rice is not detected by this test. Hence, the test in fact lends support to the alternative notion that the *sativa–rufipogon* associations are due to multiple ancestor–descendant relationships.

Wang et al. (2017) also identified 94 wild rice accessions that carried the “non-shattering” *sh4* allele (T at the functional SNP site). Again, the authors rightly point out that the presence of the “domestication” allele in wild rice may be due to shared ancestral variation or to introgression from cultivated rice. They observed that the wild accessions that carry the “domesticated” allele in the *Sh4* gene share the selective sweep with cultivated rice, with “perfect coincidence” of nucleotide diversity reduction, and interpret this as a clear signal of introgression from crop to wild rice. However, the apparent selective sweep (Wang et al., 2017) detected in wild rice is an artifact arising from their handling of the data. A similar “sweep” would be observed if we focused on any other derived allele, due to *in silico* selection of a particular variant and the associated linkage disequilibrium (see Figure 1D). The crucial observation in this test is that the *Sh4* “selective sweep” is clearly more diverse in wild rice carrying the “domestication allele” that in cultivated rice (Figure 2 in Wang et al., 2017). This is incompatible with the crop-to-wild introgression hypothesis—how can a region that had originated in cultivated rice, and was later transferred to wild rice, be more diverse in wild rice? Contrary to the interpretation of Wang et al. (2017), their analysis suggests that the diversity landscape surrounding the *sh4* “non-shattering” allele in wild rice is ancestral, and has been partially (meaning not in its full diversity) transferred into cultivated rice during domestication within a larger chromosomal block.

In summary, we show that the attempt by Choi and Purugganan (2018) to refute the conclusions of Civán et al. (2015) is invalidated by data misinterpretations that negate their claim that their reanalysis supports the introgression hypothesis. We also show that the hybrid swarm argument that attempts to explain the presence of “domestication alleles” in *O. rufipogon* (Wang et al., 2017) is similarly based on interpretation errors. Our original model for multiple domestications has recently been supported by the identification of distinct haplotypes at domestication related loci in a panel of 3,010 high-quality *O. sativa* genomes (Wang et al., 2018), and by further work of our own based on analysis of shared ancestral variation (Civán and Brown, 2018a). Both of these studies conclude that introgression from *japonica* was not necessary for establishment of the domestication phenotype in *indica*. Hence, the currently best supported hypothesis for rice domestication is consistent with polycentric origins of Asian agriculture.

## Author contributions

PC and TB contributed equally to the conception and writing of this paper.

## Conflict of interest statement

The authors declare that the research was conducted in the absence of any commercial or financial relationships that could be construed as a potential conflict of interest.

## Footnotes

^1^http://oryzasnp-atcg-irri-org.s3-website-ap-southeast-1.amazonaws.com

^2^http://cog-genomics.org/plink/1.9/
